# Evaluation of Parameters Affecting the Occurrence of Systemic Inflammatory Response Syndrome in Patients Operated on Due to Kidney Tumors

**DOI:** 10.3390/biomedicines11082195

**Published:** 2023-08-04

**Authors:** Mateusz Marcinek, Michał Tkocz, Kamil Marczewski, Robert Partyka, Leszek Kukulski, Krystyna Młynarek-Śnieżek, Bogumiła Sędziak-Marcinek, Paweł Rajwa, Adam Berezowski, Danuta Kokocińska

**Affiliations:** 1 Department of Urology, Faculty of Medical Sciences in Katowice, Medical University of Silesia, Plac Medyków 1, 41-200 Sosnowiec, Poland; 2 Department of Emergency Medicine, Faculty of Medical Sciences in Katowice, Medical University of Silesia, Francuska 20, 40-027 Katowice, Poland; 3 Department of Cardiac, Vascular and Endovascular Surgery and Transplantology, Medical University of Silesia in Katowice, Silesian Centre for Heart Diseases, Curie-Skłodowskiej 9, 41-800 Zabrze, Poland; 4 Department of Urology, Voivodeship Specialised Hospital No. 3, Energetyków 46, 44-200 Rybnik, Poland; 5 Department of Ophthalmology, Faculty of Medical Sciences in Zabrze, Medical University of Silesia, Panewnicka 65, 40-760 Katowice, Poland; 6 Department of Urology, Faculty of Medical Sciences in Zabrze, Medical University of Silesia, 3 Maja 13/15, 41-800 Zabrze, Poland; 7 Department of Urology, Medical University of Vienna, Währinger Gürtel 18-20, 1090 Vienna, Austria; 8 Beskidzkie Centrum Medyczne, Młodzieżowa 21, 43-309 Bielsko-Biała, Poland

**Keywords:** kidney tumor, systemic inflammatory response syndrome, nephrectomy

## Abstract

The application and prognostic nature of systemic inflammatory reaction syndrome (SIRS) is still being researched, as using SIRS parameters to predict patient status is cheap, efficient, fast, and easy. The study aimed to determine SIRS markers and postoperative complications occurrence in patients undergoing kidney tumor surgery, and to verify if SIRS occurrence depends on age, sex, BMI (body mass index), comorbidities, patients’ general condition before the surgery, type of surgery, intraoperative blood loss, or intraoperative ischemia time. Body temperature, heart rate, respiratory rate, and leukocyte count were measured in patients (*n* = 285) operated on due to a kidney tumor on the first (T0) and third (T3) postoperative day. Univariable and multivariable logistic regression were used to analyze the factors affecting postoperative SIRS and complications occurrence. T0: SIRS developed in patients with higher BMI, >2 ASA points, and more substantial intraoperative blood loss. T3: SIRS developed in obese or overweight patients, with >2 ASA points, significantly higher relative HR change, lower relative body temperature change, respiratory rate, and leukocyte count. BMI values, preoperative general health status, and the amount of intraoperative blood loss in patients undergoing surgery due to a kidney tumor can contribute to SIRS occurrence. Patient’s sex, age, tumor size, type of surgery, operated side, and time of intraoperative ischemia do not affect SIRS occurrence.

## 1. Introduction

Renal malignancies account for 2–3% of all neoplasms observed in adults, while cancers derived from renal tissue comprise 90% of all malignant tumors [1].

Environmental factors seem to significantly impact the incidence of kidney cancer; however, the etiology of renal tumor development is not yet fully defined. Among the etiological factors, the greatest impact on tumor development is smoking, being overweight or obese, long-term use of hypertension medication, painkillers, phenacetin, and thiazide drugs, and consumption of animal protein and coffee [2,3].

Numerous studies on systemic inflammatory reaction syndrome (SIRS) confirmed that its occurrence correlates with single or multiple organ failure development and an increase in deaths. Talmor et al. reported a prospective analysis of 2300 surgical ICU admissions during a 49-month period. Daily and cumulative multiple organ dysfunction scores and SIRS scores were recorded. In the presented study, defined end points were hospital mortality, days in the ICU, and organ dysfunction [4]. SIRS is related to longer hospitalization time [4,5], longer ICU hospitalization [6], and infection, sepsis, or severe sepsis development [7]. Comstedt et al. studied a 154 patients and found that SIRS status on admission was moderately associated with infection and strongly related to 28-day mortality [7]. The resolution of SIRS is also related to its increased duration and treatment results (*n* = 702) [8]. Stephenson et al. studied a total of 179 patients and showed that SIRS patients required more therapeutic interventions and surgical interventions, intensive treatments, and longer hospital stays, as well as experiencing more frequent deaths [6]. Using SIRS to predict patient status is cheap, efficient, fast, and easy. SIRS can be diagnosed when two or more of the following four criteria are met: (1) body temperature <36 °C (hypothermia) or >38 °C (fever); (2) heart rate >90/min (tachycardia); (3) respiratory rate >20/min (tachypnea) or pCO_2_ < 32 mmHg; (4) leukocytes count <4 G/L (leukopenia) or >12 G/L (leukocytosis) or immature neutrophils count ≥10% (bandemia) [4,5,6,7,8]. Evaluation of SIRS criteria allows for quickly determining the increased risk of serious complications. This enables adequate response with appropriate therapy, increased vigilance, or further extended laboratory tests [9]. The combined effect of noradrenaline and cortisol, and decreased IL-12 concentration, leads to Th1 and Th2 imbalance, namely a decrease in the activity of Th1 and an increase in the activity of Th2 [9]. That in turn causes a change in the concentrations of individual cytokines produced by them, and thus, a decrease in cellular immunity [9]. Surgeries on patients with ischemia-reperfusion syndrome have a significant impact on SIRS development increasing oxidative stress and inflammatory processes [9]. SIRS is associated with the development of single or multiple organ failure [9,10]. Since SIRS criteria monitoring is simple and enables excluding dangerous complications, SIRS could be widely used in the postoperative period, because it very well identifies a clinical condition associated with a systemic inflammatory reaction [10].

The presented study aimed to determine markers of SIRS and postoperative complications occurrence in patients undergoing kidney tumor surgery. We aimed to verify whether the SIRS occurrence depends on the following factors: age, sex, BMI (body mass index), comorbidities (such as diabetes and hypertension), patients’ general condition before the surgical procedure, tumor size, operated side of the body, type of surgery (nephrectomy or organ-sparing surgery), intraoperative blood loss, or intraoperative ischemia time.

## 2. Materials and Methods

### 2.1. Study Group and Design

The study included 285 patients operated on due to kidney tumors in the Clinical Department of Urology and Urological Oncology of the 5th Specialist Hospital in Sosnowiec in 2018–2020. The study was approved by the Ethics Committee of the University of Silesia Medical Center in Katowice (No. PCN/022/KB1/73/20, date of approval: 13 October 2020) and was conducted according to the Declaration of Helsinki guidelines. Each patient was informed about the purpose, methods, risks, and benefits of the study and gave written consent to participate in the study.

The study included patients with clinically diagnosed and histopathologically confirmed kidney cancers. Patients operated on in a life-saving mode, under 18 years of age, diagnosed with other cancer(s), displaying inflammatory or autoimmune symptoms, and who did not agree to participate were excluded from the study.

Before the operation, patients were interviewed for present or past comorbidities and current medication use and examined by a urologist and anesthesiologist. Blood and urine were collected for biochemical tests. The abdominal cavity and pelvis minor were examined using ultrasonography and computed tomography or magnetic resonance imaging with contrast. Patients were evaluated by an anesthesiologist using the ASA (American Society of Anesthesiology) scale and qualified for the surgery. Depending on the examination and the imaging results, patients underwent either a nephrectomy or organ-sparing surgery [11,12,13,14].

After the operation, patients were classified according to the TNM system used for the clinical staging of tumors and assigned to relevant groups. On the first (T0) and third (T3) postoperative day, SIRS parameters, i.e., body temperature, heart rate, respiratory rate, and leukocyte count, were measured.

### 2.2. Statistical Methods

The normality of data distribution was assessed by the Shapiro–Wilk test and the quantile-quantile plot. Normally distributed interval data were presented as mean (SD). Non-normally distributed interval data were presented as median and lower and upper quartile—Me (Q_1_; Q_3_). Qualitative data were presented as numbers and percentages. The dichotomous variables comparison was done using χ2 test or the χ2 test with the Yates correction if the expected number was <5, or in case the data failed to meet Cochran’s assumptions, with the Fisher’s test. The comparison of two variables with normal or log- transformed (due to skewness) interval data was done using the Student’s t-test for independent variables. The comparison of two variables with data deviating from the normal distribution was done using the Mann-Whitney U test. The analysis of variance with repeated measures and contrast analysis was used for dependent variables over time and comparisons between groups with and without SIRS.

Factors affecting the SIRS or complications occurrence after the procedure were analyzed using a univariable and multivariable logistic regression. The data for the multivariable logistic regression comprised the variables marked as significant during the univariable analysis. The results were presented as odds ratios (OR) with a 95% confidence interval (CI) and corresponding statistical significance level. Histograms and box plots were used to graphically present the results. Statistical significance was set at *p* < 0.05. All analyses were done using Statistica version 13.3 (TIBCO, Paolo Alto, USA) and R CRAN software, version 4.3.1 (R Core Team, Vienna, Austria).

## 3. Results

### 3.1. Study Group Characteristics

In total, 285 patients participated in the study, including 132 (46.3%) women and 153 (53.7%) men. The average age of the patients was 63 (11) years (range: 23–91 years), even though 49.1% of patients were above 65 years.

**Figure 1:**
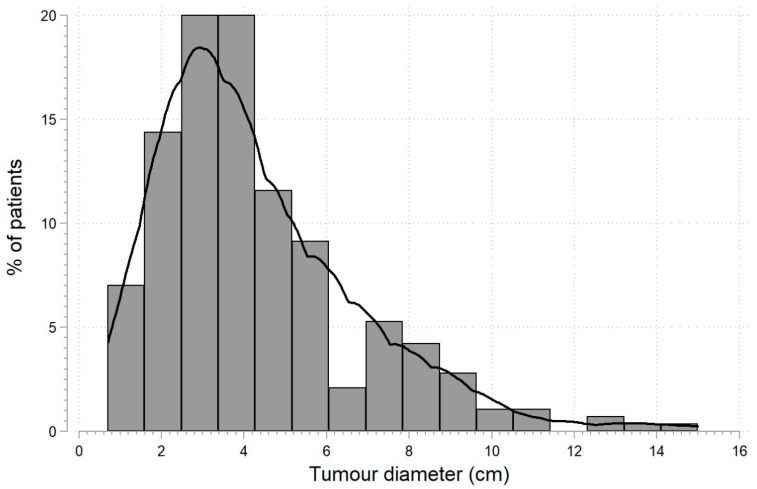
Tumor diameter distribution in patients (*n* = 285) diagnosed with kidney tumors subjected to nephrectomy or organ-sparing surgery.

Histopathological assessment recognized 248 cases of ccRCC, 26 cases of pRCC, 8 cases of chRCC, and 8 cases of oncocytoma. Surgery on the right side of the body was performed in 135 (47.4%) patients, while organ-sparing surgery in 155 (54.4%) patients. The tumor diameter ranged from 0.7–15 cm with a median of 3.7 (2.5; 5.7) cm (Figure 1). In 20 cases (7%), the patients had multifocal tumors. After the procedure, the patients were discharged from the ward on the 6th day on average (range: 3–25 days).

The average BMI of study participants was 25.5 (3.5) kg/m^2^ (range: 14–32 kg/m^2^). Overweight and obesity were diagnosed in 162 (56.8%) and 26 (9.1%) patients, respectively. Concomitant diseases were noted in 177 (62.1%) patients, including arterial hypertension in 64.6%, diabetes in 22.8%, and solitary kidney in 2.5%. Local recurrence affected 2.5% of patients.

Elevated creatinine levels were found in 26 (9.1%) patients, and 78 (27.4%) patients scored >2 on the ASA scale, indicating an increased operational risk related to serious complications or death during or after anesthesia. The average noted intraoperative blood loss was 150 (100; 210) mL, and it ranged from 50–700 mL. For patients who underwent organ-sparing surgery, the average time of intraoperative ischemia was 12 (3.9) min (range: 0–20 min). Systemic inflammatory reaction syndrome occurred in 127 (44.6%) patients on the first and in 35 (12.3%) on the third postoperative day. Postoperative complications occurred in 44 (15.4%) patients.

### 3.2. SIRS Occurrence on the First Day after Surgery Due to a Kidney Tumor

The occurrence of SIRS was not related to the sex and age of the patients, yet it depended on the operated side of the body but not on the tumor TNM classification, size, or type of the surgery, i.e., nephrectomy or organ-sparing surgery (*p* = 0.058). The time of the intraoperative ischemia and creatinine level were also not related to SIRS occurrence (Table 1). The patients who developed SIRS had a statistically significant higher BMI, so they were more often overweight or obese, but less frequently presented comorbidities.

In addition, patients who developed SIRS after surgery more often obtained >2 points on the ASA scale, suffered from more substantial intraoperative blood loss, and had longer hospitalization time.

### 3.3. Factors Influencing SIRS Occurrence on the First Day after Surgery Due to a Kidney Tumor

Univariable and multivariable logistic regression of the study participants’ results included the patient’s sex, age, and BMI, tumor size, type of surgery performed (nephron- sparing surgery (NSS) or nephrectomy), presence of comorbidities, obtaining > 2 points in the ASA classification, intraoperative blood loss, and intraoperative ischemia time. Detailed results of the univariable and multivariable analysis of the variables studied in the patients participating in the study are presented in Table 2.

The univariable analysis results showed that the odds of developing SIRS after the surgical procedure increased with the BMI (OR = 1.461), the intraoperative blood loss (OR = 1.773 for each 100 mL lost), or ASA classification > 2 (OR = 4.130) increase. Other analyzed factors, including comorbidities, did not increase the chance of SIRS occurrence after the procedure.

The multivariable analysis results confirmed the statistically significant impact on SIRS occurrence for all the factors indicated by the univariable analysis, except for BMI. The goodness of fit of the multivariable logistic regression model was moderate (R2 = 0.422).

### 3.4. SIRS Occurrence on the Third Day after Surgery Due to a Kidney Tumor

Patients who developed SIRS on the third day after surgery due to a kidney tumor had statistically significantly higher BMI, so they were more often obese or overweight, and more often scored >2 points on the ASA scale. We observed a more significant intraoperative blood loss and SIRS occurrence in these patients on the first day after the procedure. In addition, on average, patients who developed SIRS on the third postoperative day were discharged from the ward one day later. The analysis indicated that the size of multifocal tumors tended to be larger in male patients who developed SIRS on the third day after the surgery (tendency to statistical significance, *p* = 0.062)—see Table 3.

Patients who developed SIRS on the third day after the surgery also had significantly higher relative HR change, lower relative body temperature change, respiratory rate, and leukocyte count.

No statistically significant differences in SIRS occurrence on the third day after the surgery were found for sex, age, operated body side, tumor size, tumor TNM classification, type of surgery, concomitant diseases occurrence, intraoperative ischemia time, and baseline creatinine concentration (Table 3).

**Figure 2:**
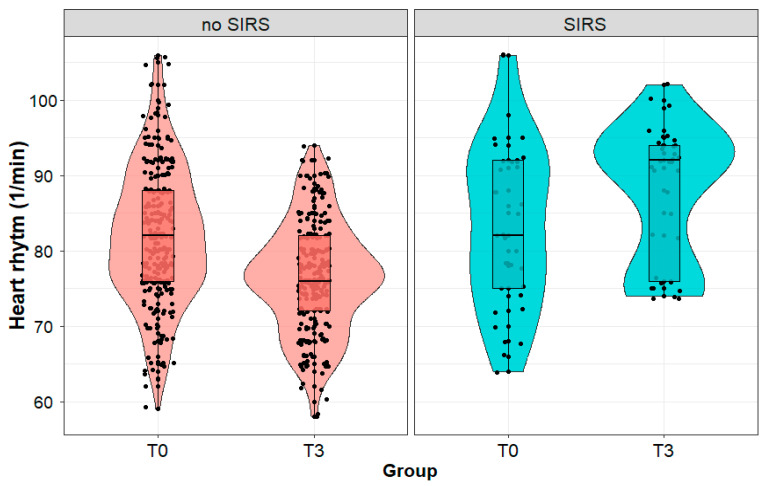
Heart rate (1/min) in patients with and without systemic inflammatory reaction syndrome (SIRS) developed on the third day after the surgery, measured immediately after (T0) and on the third day (T3) after surgery performed due to a kidney tumor. Legend: HR—heart rate.

We observed a significant increase in the pulse value in patients with SIRS (84 (11) vs. 88 (9) 1/min; *p* < 0.01) and a significant decrease (82 (9) vs. 76 (7) 1/min; *p* < 0.001) in the pulse value in patients without SIRS during the postoperative follow-up (Figure 2). Immediately after the procedure (T0), we found no significant difference (*p* = 0.682) between the patients with and without SIRS developed on the third day. Patients diagnosed with SIRS on the third day after the surgery had statistically significantly higher HR (*p* < 0.001) that day than patients without SIRS.

**Figure 3:**
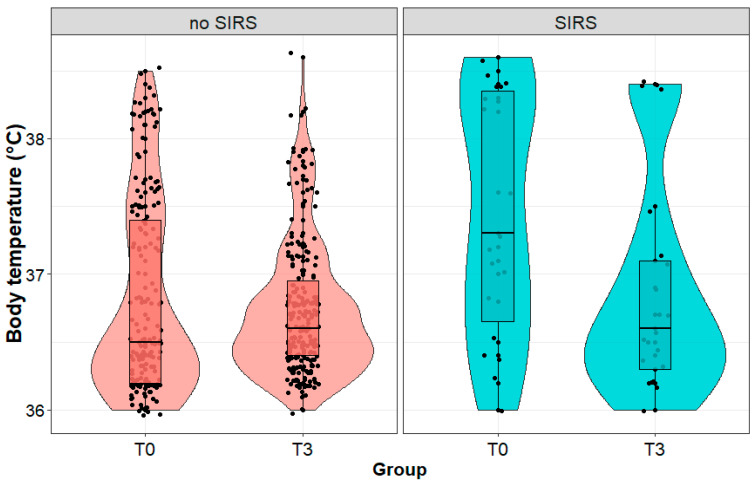
Body temperature (°C) in patients with and without systemic inflammatory reaction syndrome (SIRS) on the third day after surgery, measured immediately after (T0) and on the third day (T3) after the surgery performed due to a kidney tumor.

We observed a significant decrease (36.6 (1.1) vs. 35.8 (2.6) °C; *p* < 0.001) in body temperature in patients with SIRS and no change (*p* = 0.091) in body temperature in patients without SIRS during postoperative observation (Figure 3). We observed no significant difference (*p* = 0.493) in body temperature between the patients with and without SIRS immediately after the procedure. In contrast, on the third day after the procedure, the body temperature of patients diagnosed with SIRS was statistically significantly lower (*p* < 0.001) than in patients with SIRS on the first day.

**Figure 4:**
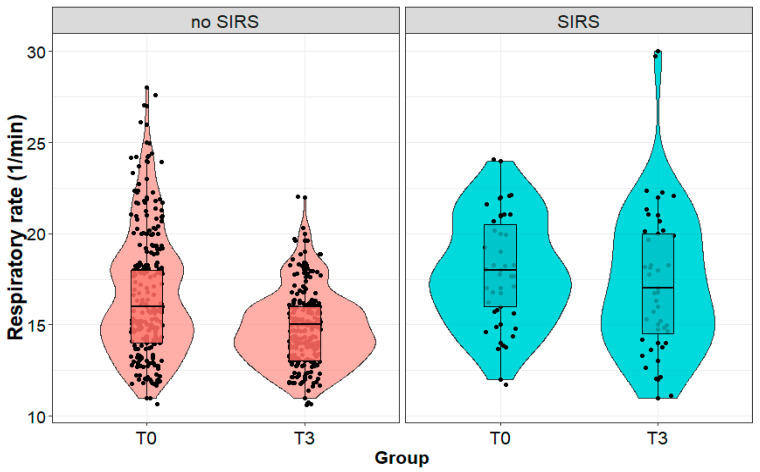
Respiratory rate (1/min) in patients with and without systemic inflammatory reaction syndrome (SIRS) on the third day after surgery, measured immediately after (T0) and on the third day (T3) after surgery performed due to a kidney tumor.

The respiratory rate of patients who developed SIRS on the third day after surgery was statistically significantly higher on the third day after surgery than in patients without SIRS (17 (4) vs. 15 (2) 1/min; *p* < 0.001), although we observed no significant difference (*p* = 0.981) between the patient groups immediately after the surgery. We observed no change (*p* = 0.372) in the respiratory rate in patients diagnosed with SIRS on the third postoperative day. On the other hand, the respiratory rate significantly decreased (*p* < 0.001) in the group of patients without SIRS (Figure 4).

**Figure 5:**
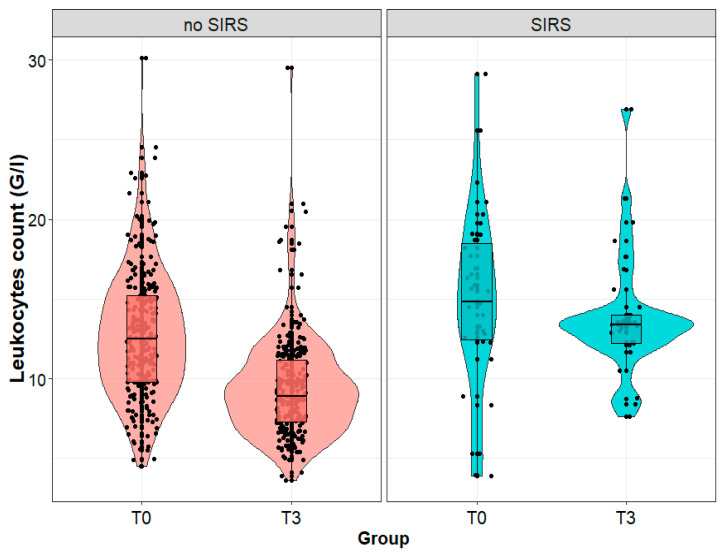
Leukocytes count (G/L) in patients with and without systemic inflammatory reaction syndrome (SIRS) on the third day after surgery, measured immediately after (T0) and on the third day (T3) after the surgical removal of the kidney tumor.

On the third day after the procedure, the leukocyte count in the serum of patients diagnosed with SIRS on the third day after the procedure was statistically significantly higher than in patients without SIRS (13.4 (12.2; 14.0) vs. 8.9 (3.6; 29.5) G/L; *p* < 0.001), although we observed no significant difference between the groups immediately after the procedure (*p* = 0.581). We noted a slight decrease in the leukocyte count in patients diagnosed with SIRS on the third day after the procedure (a statistically insignificant change), and a significant decrease in patients without SIRS (12.5 (9.8l; 15.2) vs. 8.9 (7.3; 11.2) G/L; *p* < 0.001) (Figure 5).

### 3.5. Factors Influencing SIRS Occurrence on the Third Day after Surgery Due to a Kidney Tumor

Univariable analysis showed that the odds ratio of developing SIRS on the third day after the surgery increased with increased BMI (OR = 1.374; 95%CI: 1.181–1.600; *p* < 0.001), increased intraoperative blood loss (OR = 1.768, per 100 mL; 95%CI: 1.332–2.348, *p* < 0.001), ASA classification >2 (OR = 5.079; 95%CI: 2.420–10.658; *p* < 0.001), and leukocytosis occurrence on the first postoperative day (OR = 27.385; 95%CI: 6.383–117.494; *p* < 0.001), while female sex decreased it (OR = 0.420; 95%CI: 0.193–0.913; *p* < 0.05). The rest of the analyzed factors did not change the patient’s chances of developing systemic inflammatory reaction syndrome on the third day after the surgery.

### 3.6. Complications Occurrence after Surgery Due to a Kidney Tumor

Postoperative complications occurred in 44 (15.4%) patients enrolled in the study. Patients with complications stayed in the ward longer, were more likely to have had the right side of the body operated on, were more likely to have multifocal tumors (tendency to statistical significance, *p* = 0.063), and had higher BMI values, including overweight or obesity. Additionally, patients with complications had greater intraoperative blood loss, more frequently an ASA classification > 2, higher pulse values (on the first and third day after surgery), as well as higher respiratory rate and leukocytosis on the third day after surgery.

We also observed a statistically significantly higher incidence of SIRS occurrence immediately after and on the third day after surgery, a lower relative change in body temperature, lower leukocyte count, and a greater relative change in the respiratory rate in patients with postoperative complications.

## 4. Discussion

The presented results indicate that the occurrence of SIRS on the first and third day after surgery due to a kidney tumor mainly depends on the BMI value of patients undergoing surgery. Obesity is one of the most important risk factors for developing cancer, heart disease, and metabolic disease [15], changing current medical and surgical strategies in Western societies [16]. The relationship between obesity and chronic diseases seems clear, but the relationship between obesity and the onset of SIRS is still poorly understood. The results of this study indicate that overweight/obesity measured by BMI is the strongest factor determining SIRS occurrence in patients undergoing surgery due to a kidney tumor.

The inflammatory state itself is associated with obesity and metabolic syndrome. The clinical picture of patients with insulin resistance and abdominal obesity, regarding cytokine profile, inflammatory profile, and morbidity, is similar to that observed in Gram-negative sepsis, but the severity of symptoms is lower than in sepsis [17]. Chen et al. [18] analyzed SIRS development in patients with different visceral-to-subcutaneous adipose tissue (VAT/SAT) ratios after multiple traumas. They analyzed whether adipose tissue distribution affects SIRS development in patients with multiple traumas. They found that a lower VAT/SAT ratio was associated with increased inflammatory response and poorer clinical outcomes in patients with multiple traumas. Furthermore, the VAT/SAT ratio is an independent factor providing additional information about BMI [18]. In turn, Southern et al. [19] showed a clear relationship between elevated BMI, one of the risk factors of postoperative fever, and SIRS occurrence after ureteroscopy due to nephrolithiasis. The authors studied 2746 patients who underwent 3298 URS for stone disease at Geisinger from 2008 to 2016. In addition, this study showed that the risk of SIRS significantly depended also on the sex and age of the patients, the presence of bacteria in the patient’s urine, and the duration of the surgical procedure. Older patients were more likely to have difficult hospitalization. On the other hand, female sex, longer surgery, increased BMI, and positive urine culture before surgery turned out to be significantly associated with postoperative fever/SIRS occurrence after ureteroscopy, which translated into longer hospitalization [19]. These results are consistent with the results of our study. Patients who developed SIRS on the third day after surgery due to a kidney tumor were discharged from the ward one day later on average.

Zhu et al. reported a positive correlation between the percentage of visceral fat and the stage of Fuhrman’s tumor in patients with RCC classified as T1a [20]. Ladoire et al. [21] showed that visceral obesity has a significant prognostic value in patients with advanced RCC treated with targeted therapy [21], and Steffens et al. [22] confirmed that patients with metastatic RCC (mRCC) and a higher percentage of visceral fat receiving targeted therapy achieved longer tumor-specific survival and longer overall survival [22]. On the contrary, Mano et al. [23], as one of the few, showed that neither subcutaneous adipose tissue nor visceral adipose tissue correlate with RCC stage and overall survival in patients with non-metastatic clear cell carcinoma [23]. In turn, Hakimi et al. [24] showed that overweight patients diagnosed with RCC have a better prognosis than people with normal body mass or underweight [24]. Accumulation of adipose tissue, such as high levels of SFA and TFA at the time of tumor progression, may improve the survival of mRCC patients treated with tyrosine kinase inhibitors, especially those with larger tumor burdens. Moreover, the number of increased adipose tissue components is a key prognostic factor in these patients, and hence, the accumulation of adipose tissue should be considered an important parameter for assessing the survival of patients with mRCC [25]. The mechanism by which visceral fat accumulation (VFA), subcutaneous fat accumulation (SFA), or total fat accumulation (TFA) occurs, to improve the survival of patients with mRCC, is not well understood. VFA and SFA differ by the type of adipocytes (fat cells) involved, endocrine function, lipolytic activity, and response to insulin and other hormones. The impaired or changed metabolisms (mainly glucose and lipid metabolism, primarily fatty acid synthesis and β-oxidation) are observed in cancer cells and could facilitate cell growth and proliferation. Patients with visceral obesity have been reported to have an increased risk of metabolic complications, e.g., metabolic syndrome [25]. Translational studies have shown that RCC can be induced by long-term intake of a high-fat diet, which was confirmed by pathological changes observed in histological sections [26].

Interestingly, obesity is a factor that favors a good prognosis of RCC, even though it contributes to an increased risk of RCC. For patients with organ-confined but not advanced RCC, being overweight improved their tumor-specific survival [27]. Another study also found that tumor-specific survival, but not overall survival, was significantly prolonged in patients with a higher BMI (>30 kg/m^2^) undergoing radical nephrectomy [28]. These observations partially coincide with our results presented here. Multivariable analysis showed that the comorbidities presented a protective effect (OR = 0.36) on SIRS occurrence after renal tumor surgery. In addition, it showed that people without comorbidities were almost three times more likely to develop SIRS; however, the goodness of fit of the multivariable logistic regression model was only moderate.

In the presented study, SIRS occurred in 44.6% on the first day and in 12.3% of patients on the third day after surgery. Postoperative complications were reported in 15.4%. One of the factors influencing SIRS occurrence was the higher intraoperative blood loss. Patients with heavier bleeding were by 34.8% more likely to develop SIRS. Patients who developed SIRS were also more likely to have an ASA score >2. Patients with >2 ASA score also had greater intraoperative blood loss, more frequent SIRS occurrence (94.3%) on the first postoperative day, and leukocytosis on the third postoperative day (88.6%). Every kidney-sparing surgical technique described in the literature, such as enucleation, excavation, resection of the kidney pole, extensive transverse resection, and partial ex vivo resection followed by autotransplantation, is characterized by a certain percentage of postoperative complications. Some of these techniques, such as enucleation or extracorporeal resection with autotransplantation, are performed rarely and only in special cases of large and particularly difficult-to-access tumors [29].

Intraoperative blood loss or postoperative bleeding has been associated with SIRS. Despite the relatively low incidence of bleeding after partial nephrectomy, estimated at 4.2–6% for laparoscopic partial nephrectomy [30], 6% for open procedures, and 8.1% for robotic procedures [31], postoperative bleeding remains one of the most serious complications, especially in the case of centrally located tumors [32]. Several studies have examined various factors associated with hemorrhage after partial nephrectomy, including patient demographics, surgical method, and tumor parameters [33]. Van Poppel et al. [34], in their study of open partial nephrectomy in 76 patients, suggested that larger tumor size and its central location correlate with an increased risk of postoperative hemorrhage. Similarly, Ramani et al. [33] showed that the frequency of postoperative bleeding was higher in patients with central tumors and deeper infiltration. In our study, the univariable analysis indicated that for each 100 mL lost amount of intraoperative blood the odds of developing SIRS after the procedure increased by 77.3%.

The presented results showed that patients who developed SIRS on the third day after surgery more often scored >2 on the ASA scale and were discharged from the ward one day later on average. It can be concluded that SIRS occurrence affects the length of hospitalization. Uchida et al. analyzed preoperative and intraoperative SIRS risk factors after ureteroscopy, as infectious complications are one of the most worrying problems of urolithiasis treatment [35]. Uchida et al. [35] and Martov et al. [36], in their independent studies under the CROES URS Global Study, showed that female sex, elevated ASA, high stone burden, Crohn’s disease, and cardiovascular disease are significant risk factors for postoperative urinary tract infection, fever, and SIRS in patients with negative baseline urine cultures [35,36]. The analyzes of preoperative and intraoperative risk factors for SIRS development after surgical procedures used in urology and other types of surgery are abundantly available. However, no consensus among researchers on the risk factors predicting SIRS has been achieved due to the complexity of pathophysiology and surgical factors. According to Moses et al. [37], age, sex, BMI, ASA score, and duration of surgery are not risk factors for SIRS after percutaneous nephrolithotomy [37]. On the other hand, an analysis by Akdeniz et al. [38] showed that diabetes, acute phase proteins (CRP), platelet-to-lymphocyte ratio, neutrophil-to-lymphocyte count, urinary white blood cell count, stone size, mean ASA score, type of surgery, duration of surgery, mean hemoglobin drop, length of hospital stay, blood transfusion, and complication rate were associated with SIRS development after percutaneous nephrolithotomy, but age, gender, BMI, and location of stones were not [38]. Takenaka et al. [39], in turn, indicated that postoperative monitoring of acute phase parameters (IL–6 and CRP) and SIRS parameters is very important, as they correlate well with the intensity of surgical stress and the length of hospitalization. Haga et al. [40] studied the incidence of SIRS and multiple organ dysfunction in patients undergoing gastrointestinal surgery. The results showed that the length of SIRS or the number of positive SIRS criteria after surgery significantly correlated with parameters of surgical stress (blood/weight loss and duration of surgery) and CRP value. The systemic inflammatory reaction syndrome, which persisted or reappeared after the third postoperative day, was an early sign of postoperative complications. Researchers concluded that SIRS is a useful criterion for diagnosing postoperative complications. Becher et al. [41], studying the impact of the inflammatory response on the outcome of patients undergoing emergency colorectal surgery, concluded that failure to regulate the body’s systemic inflammatory response was the leading cause of death in patients undergoing the unscheduled surgery and patients with SIRS or sepsis who underwent surgery shorter than 2.5 h had fewer postoperative complications. Their results further support the importance of timely surgical intervention with the best possible control of tissue manipulation, potentially reducing inflammation. Smajic et al. [42] showed that SIRS incidence in patients subjected to unscheduled surgery (86.7%) tended to decrease gradually during the postoperative period, to 60% 24 h after surgery and 40% 72 h after surgery [42]. They explained the high SIRS incidence before surgery by the already-present initial inflammatory reaction, forcing the scheduled procedure. They observed a high rate of postoperative SIRS, which gradually decreased as the root cause of the inflammatory response subsided. The following residual effects of SIRS intertwined with the effect of the surgical procedure acted as an additional stress stimulus. The SIRS criteria are a postoperative reflection of the activated inflammatory cascade that tends to decrease. Smajic et al. [42] also showed that the SIRS score correlates with the hospitalization time and slightly less with the treatment outcome, which can be explained by the resolving inflammation cascade within 72 h after surgery [42]. These results coincide with the presented observations regarding the relationship between SIRS occurrence on the third day after surgery and the hospitalization time.

In this study, SIRS occurrence was not related to the time of intraoperative ischemia. The mean intraoperative ischemia time in patients undergoing organ-sparing surgery was 12 ± 3.9 min (range: 0–20 min). Nephrectomy procedures should always aim at minimizing ischemic time. Research evidence and patients’ observations indicate that a time interval of up to 20–25 min is the most accurate cut-off point for ischemic patients who do not develop a short-term and long-term decline in renal function after partial nephrectomy, depending on the amount of renal tissue saved. According to the latest studies, good clinical practice (i.e., achieving relatively short periods of warm ischemia) means that ischemic time is not the strongest factor influencing long-term renal function, since renal filtration depends on the quality and quantity of preserved renal parenchyma. Despite the lack of consistent data indicating a significant relationship between intraoperative ischemia and end-stage renal disease in patients with both kidneys, warm ischemic time remains a strong predictor of acute kidney injury and the need for dialysis after partial nephrectomy in solitary kidneys [43]. Thompson et al. [44] reported that warm ischemia longer than 25 min was associated with a 2.3-fold increased risk of chronic kidney disease after partial nephrectomy [44]. These data suggest that the loss of function due to limited warm ischemia is marginal; however, prolonging the duration of warm ischemia may be detrimental.

## 5. Conclusions

BMI values, preoperative general health status measured with the ASA scale, and the amount of intraoperative blood loss in patients undergoing surgery due to a kidney tumor can contribute to SIRS occurrence. On the other hand, the patient’s sex, age, tumor size, type of surgery, operated side, and time of intraoperative ischemia do not affect SIRS occurrence.

## Figures and Tables

**Table 1:** Basic statistics describing patients with and without systemic inflammatory reaction syndrome (SIRS) on the first day after surgery due to a kidney tumor. Results are presented as mean (SD) or median (lower quartile; upper quartile).

Study Variable	SIRS *n* = 127 (44.6%)	No SIRS *n* = 158 (55.4%)	*p*
Sex M/F, *n* (%)	67/60 (52.8/47.2)	86/72 (54.4/45.6)	0.78
Age, years	62 (11)	64 (12)	0.25
Age ≥ 65 years, *n* (%)	56 (44.1)	84 (53.2)	0.13
Comorbidities, *n* (%)	68 (53.5.7)	109 69.0)	<0.01
Hypertension, *n* (%)	83 (65.4)	101 (63.9)	0.80
T2DM, *n* (%)	34 (26.8)	31 (19.6)	0.15
BMI, kg/m^2^	27.3 (2.9)	24.0 (3.2)	<0.001
Overweight, *n* (%)	91 (71.7)	71 (44.9)	
			<0.001
Obesity, *n* (%)	21 (16.5)	5 (3.3)	
ASA > 2, *n* (%)	54 (42.5)	24 (15.2)	<0.001
Solitary kidney, *n* (%)	2 (1.6)	5 (3.2)	0.47
Cancer local recurrence, *n* (%)	5 (3.9)	2 (3.2)	0.15
Operated side: R/L, *n* (%)	70/57 (55.1/44.9)	65/93 (41.1/58.9)	<0.05
Tumor diameter, cm	4.0 (2.5; 5.0)	3.6 (2.5; 6.0)	0.76
Multifocal tumor, *n* (%)	12 (9.4)	8 (5.1)	0.17
TNM: T1a, *n* (%)	78 (61.4)	97 (61.4)	
TNM: T1b, *n* (%)	37 (29.1)	46 (29.1)	
TNM: T2a, *n* (%)	7 (5.5)	10 (6.3)	0.95
TNM: T2b, *n* (%)	3 (2.4)	4 (2.5)	
TNM: T3a, *n* (%)	2 (1.6)	1 (0.7)	
Surgery type: NSS/nephrectomy, *n* (%)	77/50 (60.6/39.4)	78/80 (49.4/50.6)	0.058
Intraoperative blood loss, mL	180 (120; 250)	120 (80; 190)	<0.001
Ischemia time, min	12.1 (4.0)	11.9 (3.8)	0.71
Creatinine, mg/dL	1.0 (0.8; 1.1)	1.0 (0.9; 1.1)	0.77
Creatinine > 1.1/1.4 mg/dL *, *n* (%)	10 (7.9)	16 (10.1)	0.51
HR, 1/min	85 (11)	80 (7)	<0.001
Postoperative body temperature, °C	36.4 (1.1)	36.5 (0.7)	0.47
Respiratory rate, 1/min	18 (4)	16 (3)	<0.001
Leukocytes, G/L	14.7 (12.5; 17.0)	11.2 (9.1; 13.6)	<0.001
Leukocytes > G/L, *n* (%)	109 (85.8)	98 (62.0)	<0.001
Hospitalization time, days	6 (5; 7)	5 (4; 7)	<0.05

Legend: * The value 1.1 mg/mL applies to women, and the value 1.4 mg/mL applies to men. Abbreviations: ASA—American Society of Anesthesiology system, assessing patients’ general condition and the risk of severe complications or death during or after anesthesia; BMI—body mass index; HR—heart rate; F—female; M—male; NSS—nephron-sparing surgery; SIRS—systemic inflammatory response syndrome; TNM—International Union Against Cancer (UICC) renal tumor classification system; T1a—tumor limited to kidney, <4 cm; T1b—tumor limited to kidney, >4 cm and ≤7 cm; T2a—tumor limited to kidney, >7 cm and ≤10 cm; T2b—tumor limited to kidney, >10 cm; T3a—tumor infiltrating the renal vein or its branches, the pyelocaliceal system, the perirenal fat, or perirenal sinus fat, but not beyond Gerota’s fascia or the adrenal gland.

**Table 2:** Results of univariable and multivariable logistic regression of factors influencing the occurrence of systemic inflammatory reaction syndrome on the first day after the procedure performed due to a kidney tumor.

Study Variable	Univariable Analysis		Multivariable Analysis	
	OR	±95% CI	OR	95% CI
Sex F vs. M	1.070	0.668–1.712		
Age	0.988	0.968–1.008		
Age ≥ 65 years	0.695	0.433–1.114		
Comorbidities	0.518 **	0.318–0.843	0.359 *	0.168–0.777
BMI	1.461 ^#^	1.315–1.623		
Obesity/overweight	8.056 ^#^	4.310–15.057	4.998 ^#^	2.246–11.122
ASA > 2	4.130 ^#^	2.355–7.243	7.205 ^#^	3.221–16.001
Tumor diameter	0.978	0.888–1.077		
NSS vs. nepherctomy	1.579	0.982–2.541		
Intraoperative blood loss (per 100 mL)	1.773 ^#^	1.351–2.326	2.471 ^#^	1.695–3.603
Ischemia time	1.016	0.935–1.103		
HR	1.713 ^#^	1.306–2.246	2.143 ^#^	1.428–3.216
Respiratory rate	3.607 ^#^	2.362–5.511	3.211 ^#^	1.808–5.702
Leukocytes > G/L	3.707 ^#^	2.042–6.729	7.705 ^#^	3.221–18.428

Legend: * *p* < 0.05; ** *p* < 0.01; # *p* < 0.001; Abbreviations: ASA—American Society of Anesthesiology system assessing patients’ general condition and the risk of severe complications or death during or after anesthesia; BMI—body mass index; CI—confidence interval; HR—heart rate; F—female; M—male; NSS—nephron-sparing surgery; OR—odds ratio.

**Table 3:** Basic statistics describing patients with and without systemic inflammatory reaction syndrome (SIRS) on the third day after surgery due to a kidney tumor. Results are presented as mean (SD) or median (lower quartile; upper quartile).

Study Variable	SIRS *n* = 35 (12.3%)	No SIRS *n* = 250 (87.7%)	*p*
Sex M/F, *n* (%)	25/10 (71.4/28.6)	128/122 (51.2/48.8)	0.06
Age, years	62 (11)	63 (12)	0.68
Age ≥ 65 years, *n* (%)	14 (40.0)	126 (50.4)	0.25
Comorbidities, *n* (%)	19 (54.3)	158 (63.2)	0.31
Hypertension, *n* (%)	21 (60.0)	163 (65.2)	0.55
T2DM, *n* (%)	12 (34.3)	53 (21.2)	0.084
BMI, kg/m^2^	27.8 (2.4)	25.1 (3.5)	<0.001
Overweight, *n* (%)	28 (80.0)	134 (53.6)	
			<0.001
Obesity, *n* (%)	5 (14.3)	21 (8.4)	
ASA > 2, *n* (%)	21 (60.0)	57 (22.8)	<0.001
Solitary kidney, *n* (%)	1 (2.9)	6 (2.4)	1.00
Cancer local recurrence, *n* (%)	2 (5.7)	5 (2.0)	0.21
Operated side: R/L, *n* (%)	20/15 (57.1/42.9)	115/135 (46.0/54.0)	0.22
Tumor diameter, cm	4.0 (3.0; 6.0)	3.5 (2.5; 5.5)	0.11
Multifocal tumor, *n* (%)	7 (20.0)	13 (5.2)	<0.01
TNM: T1a, *n* (%)	21 (60.0)	154 (61.6)	
TNM: T1b, *n* (%)	7 (20.0)	76 (30.4)	
TNM: T2a, *n* (%)	4 (11.4)	13 (5.2)	0.19
TNM: T2b, *n* (%)	2 (5.7)	5 (2.0)	
TNM: T3a, *n* (%)	1 (2.9)	2 (0.8)	
Surgery type: NSS/nephrectomy, *n* (%)	16/19 (45.7/54.3)	139/111 (55.6/44.4)	0.27
Intraoperative blood loss, mL	220 (150; 250)	140 (100; 200)	<0.001
Ischemia time, min	12.3 (4.4)	12.0 (3.9)	0.74
Creatinine, mg/dL	1.0 (0.8; 1.2)	1.0 (0.8; 1.1)	0.23
Creatinine > 1.1/1.4 mg/dL *, *n* (%)	5 (14.3)	21 (8.4)	0.26
Δ HR, %	6.3 (16.0)	–5.7 (12.6)	<0.001
Δ body temperature, %	–2.1 (7.4)	0.7 (1.9)	<0.05
Δ respiratory rate, %	–1.9 (23.7)	–8.7 (18.3)	0.11
Δ leukocytes, %	–12.1 (–30.0; 15.4)	–26.0 (–39.3; –11.1)	<0.01
SIRS_T0_, *n* (%)	33 (94.3)	94 (37.6)	<0.001
Leukocytes_T0_ > G/L, *n* (%)	29 (82.9)	178 (71.2)	0.15
Leukocytes_T3_ > G/L, *n* (%)	31 (88.6)	79 (31.6)	<0.001
Hospitalization time, days	7 (5; 8)	6 (4; 6)	<0.001

Legend: Δ—relative parameter change; * The value 1.1 mg/mL applies to women, and the value 1.4 mg/mL applies to men. Abbreviations: ASA—American Society of Anesthesiology system assessing patients’ general condition and the risk of severe complications or death during or after anesthesia; BMI—body mass index; HR—heart rate; F—female; M—male; NSS—nephron-sparing surgery; SIRS—systemic inflammatory response syndrome; TNM—International Union Against Cancer (UICC) renal tumor classification system; T0—the first day after surgery; T1a—tumor limited to kidney, <4 cm; T1b—tumor limited to kidney, >4 cm and ≤7 cm; T2a—tumor limited to kidney, >7 cm and ≤10 cm; T2b—tumor limited to kidney, >10 cm; T3—the third day after surgery; T3a—tumor infiltrating the renal vein or its branches, the pyelocaliceal system, the perirenal fat, or perirenal sinus fat, but not beyond Gerota’s fascia or the adrenal gland.

## Data Availability

The data presented in this study are available on request from the corresponding author. The data are not publicly available due to legal and ethical reasons.
